# Engagement and Nonusage Attrition With a Free Physical Activity Promotion Program: The Case of 10,000 Steps Australia

**DOI:** 10.2196/jmir.4339

**Published:** 2015-07-15

**Authors:** Diana Guertler, Corneel Vandelanotte, Morwenna Kirwan, Mitch J Duncan

**Affiliations:** ^1^ Institute of Social Medicine and Prevention University Medicine Greifswald Germany; ^2^ School of Human Health and Social Sciences Physical Activity Research Group Rockhampton Australia; ^3^ DZHK (German Centre for Cardiovascular Research) Greifswald partner site Greifswald Germany; ^4^ School of Science and Health University of Western Sydney Sydney Australia; ^5^ School of Medicine & Public Health, Priority Research Centre for Physical Activity and Nutrition Faculty of Health and Medicine The University of Newcastle Newcastle Australia

**Keywords:** physical activity, Internet, engagement, smartphone

## Abstract

**Background:**

Data from controlled trials indicate that Web-based interventions generally suffer from low engagement and high attrition. This is important because the level of exposure to intervention content is linked to intervention effectiveness. However, data from real-life Web-based behavior change interventions are scarce, especially when looking at physical activity promotion.

**Objective:**

The aims of this study were to (1) examine the engagement with the freely available physical activity promotion program 10,000 Steps, (2) examine how the use of a smartphone app may be helpful in increasing engagement with the intervention and in decreasing nonusage attrition, and (3) identify sociodemographic- and engagement-related determinants of nonusage attrition.

**Methods:**

Users (N=16,948) were grouped based on which platform (website, app) they logged their physical activity: Web only, app only, or Web and app. Groups were compared on sociodemographics and engagement parameters (duration of usage, number of individual and workplace challenges started, and number of physical activity log days) using ANOVA and chi-square tests. For a subsample of users that had been members for at least 3 months (n=11,651), Kaplan-Meier survival curves were estimated to plot attrition over the first 3 months after registration. A Cox regression model was used to determine predictors of nonusage attrition.

**Results:**

In the overall sample, user groups differed significantly in all sociodemographics and engagement parameters. Engagement with the program was highest for Web-and-app users. In the subsample, 50.00% (5826/11,651) of users stopped logging physical activity through the program after 30 days. Cox regression showed that user group predicted nonusage attrition: Web-and-app users (hazard ratio=0.86, 95% CI 0.81-0.93, *P*<.001) and app-only users (hazard ratio=0.63, 95% CI 0.58-0.68, *P*<.001) showed a reduced attrition risk compared to Web-only users. Further, having a higher number of individual challenges (hazard ratio=0.62, 95% CI 0.59-0.66, *P*<.001), workplace challenges (hazard ratio=0.94, 95% CI 0.90-0.97, *P*<.001), physical activity logging days (hazard ratio=0.921, 95% CI 0.919-0.922, *P*<.001), and steps logged per day (hazard ratio=0.99999, 95% CI 0.99998-0.99999, *P*<.001) were associated with reduced nonusage attrition risk as well as older age (hazard ratio=0.992, 95% CI 0.991-0.994, *P*<.001), being male (hazard ratio=0.85, 95% CI 0.82-0.89, *P*<.001), and being non-Australian (hazard ratio=0.87, 95% CI 0.82-0.91, *P*<.001).

**Conclusions:**

Compared to other freely accessible Web-based health behavior interventions, the 10,000 Steps program showed high engagement. The use of an app alone or in addition to the website can enhance program engagement and reduce risk of attrition. Better understanding of participant reasons for reducing engagement can assist in clarifying how to best address this issue to maximize behavior change.

## Introduction

Insufficient physical activity has been identified as the fourth-leading risk factor for global mortality [1]. Previous research has shown that Web-based behavior change interventions can be effective in increasing physical activity [2,3]. The Internet is a promising tool to deliver complex, individualized, and tailored interventions while reaching a large part of the population at lower cost than face-to-face interventions [4]. However, maintaining interest of the participants in the intervention over time has been reported as a main challenge of Internet-delivered interventions [5]. Web-based intervention studies typically suffer from high nonusage attrition (ie, not all participants use or keep using the intervention as intended by the developers) [6]. Most often, website log-ins, a frequently used indicator of engagement and intervention exposure, decrease rapidly over time [7,8]. This makes it difficult to measure intervention effects because participants receive different doses of the intervention content [5]. For example, in a Web-based weight loss study [9], only 64% of the intervention group actually used the intervention at least once. This is important because the level of exposure to intervention content has been linked to intervention effectiveness [10,11]. In addition to nonusage attrition, Web-based interventions studies suffer also from high dropout attrition (ie, participants are lost to follow-up). For Web-based physical activity interventions, reported dropout attrition rates vary between 0% and 62% [2,12].

Eysenbach [5] calls for a science of attrition to systematically examine attrition rates, engagement measures, and associated variables. Thus far, research on attrition has been done primarily in the context of controlled trials [6,13]. However, to evaluate the real public health impact of an intervention there is a need to examine effectiveness and reach of the target population after implementation to real-life settings [14]. Findings of nonusage rates from controlled settings may not translate to real-life settings. In efficacy trials, participants usually have gone through a rigorous screening process to determine eligibility and therefore include a selected group of participants that is likely more motivated to use the intervention compared to those not undergoing this screening. Further, people could be more committed to the study because of the formal structure of the trial or active recruitment, which leads to lower attrition [5,6]. This is supported by studies that find a higher percentage of intervention completers and higher website usage for trial users compared to “real-life” users of the same website [15,16]. Although dropout and nonusage attrition have been described in relation to commercially available websites [17,18], few studies describe similar patterns in freely accessible interventions, especially in regard to physical activity [15].

Several intervention characteristics have shown to enhance engagement and/or decrease attrition of interventions including the provision of personally tailored content, interactive components, social networking, and reminders [19-22]. Besides characteristics of the intervention itself, personal characteristics of the users and the degree of engagement with the intervention may affect nonusage attrition [15,20,23]. Attempts to promote physical activity via smartphone technology appear to be promising because it increases the convenience of accessing the intervention and engaging in self-monitoring [24]. Within controlled trials, there is some evidence that using smartphone apps can enhance engagement, decrease attrition, and increase efficacy of Web-based interventions [25-27]. However, thus far there is no knowledge about how smartphone apps can enhance engagement with Web-based interventions in real-life settings.

The aim of this study is to examine engagement with a Web-based physical activity intervention in real life because findings from controlled settings may not translate into real-life settings. Therefore, this study examines engagement and nonusage attrition in the freely available 10,000 Steps Australia program [20,28], which aims to promote physical activity through the use of pedometers and a website. A second aim is to examine whether use of a smartphone app is associated with reduced nonusage attrition and increased engagement with the intervention. Third, we aim to identify sociodemographic- and engagement-related determinants of nonusage attrition.

## Methods

### Intervention Program

The 10,000 Steps program is a freely available physical activity promotion program that encourages users to record and monitor their physical activity using pedometers. It was initially developed as a whole-community multilevel program based on the socioecological framework. Further information on the development of the program has been reported elsewhere [20,28]. A main feature of the program is an online step log that allows users to enter and monitor their daily physical activity levels based on pedometer steps or time spent in physical activity. This feature is available to users both on the website and as a smartphone app. Further, users are able to join individual challenges where they choose from a monthly updated selection of goals and receive graph- and text-based feedback of their progress (individual challenge). When users are recruited via their workplace, they may participate in team-based workplace challenges. These usually last longer than 1 month and the workplace is responsible to set the team challenges (workplace challenge). Further, users are able to use a discussion forum and virtual walking buddies with whom they can share progress.

### Smartphone App

In addition to the opportunity to use the program via the 10,000 Steps website, there is also a smartphone app available on the iOS mobile platform [27]. Initially designed to allow users to enter their daily physical activity, users are now also able to join and view progress of challenges. Data from the smartphone app are synchronized with their activities recorded on the website. Data from a case-matched control trial [27] indicated that use of the 10,000 Steps app increased the number of days physical activity was logged and the likelihood to log more than 10,000 steps per day in a sample of 10,000 Steps users. Excluding participants from the controlled trial, the app had a total of 35,761 downloads as of April 30, 2014. One-third (33.12%, 11,845/35,761) of those that downloaded the app also used it to log physical activity.

### Data Collection and Extraction

Data were extracted from the database of the 10,000 Steps program for users aged at least 18 years, who registered between July 8, 2013 and April 30, 2014, and who logged physical activity for at least 1 day in this period either through the website or the smartphone app (N=17,590). Although the website and app were available before July 8, 2013, this date was chosen as the start date for data extraction because prior to this date the available version of the app had reduced functionality; specifically, it did not allow users to join or view progress of challenges. No major changes in app functionality have occurred since this date, only minor updates have happened. Therefore, this study was delimited to users who registered during this 297-day period between July 8, 2013 and April 30, 2014 excluding those users who registered before this time. Thus, the maximum membership length in this study was 297 days. Website and app usage data were automatically recorded while using the 10,000 Steps program. When registering with the 10,000 Steps program, participants provided informed consent for usage of their data for research purposes.

### Measures

#### Sociodemographics, “Where Did You Hear About Us?,” and Length of Membership

Date of birth, gender, and country of residence were assessed when participants registered to the 10,000 Steps program. Users were also asked how they heard about the 10,000 Steps program (21 response options were provided, including different types of media, friends, workplace, health professionals, and specific initiatives the program was advertised in). Length of membership was calculated as the number of days between the date of registration for the program and April 30, 2014.

#### Engagement Parameters

Engagement was defined as the duration and frequency of involvement with the program. Four measures of engagement were used: (1) the duration of program use calculated as the number of calendar days from the first to the last time the physical activity log was used, (2) the number of individual challenges initiated, (3) the number of workplace challenges initiated, and (4) the total number of days physical activity was recorded in the step log (both website and app). The number of individual and workplace challenges participated in was determined from the 10,000 Steps database, which encompassed website and app usage information.

The total number of days of physical activity recorded in the step log and duration of program usage differed. For example, a user may have used the program for 50 days (time from first to last step log), but only logged steps on 30 occasions during this time.

#### Nonusage Attrition

Duration of program usage was also applied as an indicator of nonusage attrition. Participants were coded as “nonusage attrition was observed” when they did not log physical activity for at least 14 days [29] (ie, there were ≥14 days between their last physical activity log and the end of the observation period). All other users were coded as “nonusage attrition was not observed.” For example, when a participant first logged steps on the 10,000 Steps platform on October 1, 2013 and the last time on December 1, 2013, then nonusage attrition was deemed to have occurred after 62 days of use.

#### Physical Activity

Users’ mean steps per day were determined by dividing the total number of steps logged by the number of days steps were logged for. If users’ mean of logged steps per occasion was more than 20,000, this was truncated to 20,000 steps [30,31]. Truncation was performed for 928 participants.

### Data Analysis

#### Overview

The program allows participants to retrospectively log steps in case they started to use a pedometer before registering with the program. Users were excluded from data analysis when they logged physical activity for more than 7 days prior to their start of their membership (n=617) and when inconsistencies in data were detected; that is, users logged on average less than 100 steps per day (n=16) or logged physical activity on more days than it would be possible based on their duration of program use (n=9). The final number of users included in the analysis of user characteristics and engagement was N=16,948 and is referred to as the overall sample. Attrition analysis was based on a subsample of users as described subsequently. Based on the platform used to log steps, 3 groups were defined: Web-only users who logged steps solely via the 10,000 Steps website (83.87%, 14,215/16,948), app-only users who logged steps solely via the 10,000 Steps smartphone app (8.56%, 1451/16,948), and Web-and-app users who logged steps via the 10,000 Steps website and the smartphone app (7.56%, 1282/16,948).

#### User Characteristics and Intervention Engagement

First, means and standard deviations for sociodemographics, engagement parameters, and logged physical activity were calculated to describe the overall sample and the 3 user groups.

To assess which personal characteristics of 10,000 Steps users may facilitate choosing the app over the website, differences between the user groups regarding sociodemographics were analyzed. Further, user groups were compared regarding engagement parameters and logged physical activity. Group comparisons were performed using 1-way ANOVAs with Bonferroni-corrected post hoc comparisons (for continuous variables) and chi-square tests (for categorical variables).

Because engagement parameters are likely to depend on sociodemographics, length of membership, and physical activity level, we also examined the effect of user group on the engagement parameters using linear regression adjusting for those variables. Four regression analyses were fitted, each using an engagement parameter (duration of usage, individual challenges, workplace challenges, physical activity log days) as the dependent variable. User group was used as the independent variable along with age, gender, country of residency, length of membership, and physical activity as covariates. Standardized regression coefficients were calculated. Effect sizes (η^2^) were calculated for linear regression because even small differences tend to reach significance with high numbers of participants such as in our study. According to Cohen [32], the minimum criterion for at least a small effect was η^2^>.01. Therefore, in this study, differences were considered meaningful when effect sizes reached these thresholds. Further, duration of usage, number of individual and workplace challenges started, and number of days physical activity was logged for were presented for different membership lengths.

#### Nonusage Attrition

Survival analysis was used to examine differences in nonusage attrition between groups. Nonusage attrition was examined over the first 3 months after registration to the program and was limited to a subsample of users (n=11,651) who were a 10,000 Steps member for at least 3 months (90 days) at the time of data extraction (April 30, 2014). This was done to ensure that all participants had the same chance to use the program and to enable comparability with other published attrition curves [5,15,33]. This means that users were included even if they only logged steps for a single day, but had been a member for 3 months. The duration of program use was used as the time variable. The event variable was coded as specified in the Measures section with 1=nonusage attrition observed and 0=nonusage attrition not observed. Kaplan-Meier survival curves showing the proportion of users surviving over time and quartiles of survival time were estimated by user group. The equality of the survivor functions was tested with a log-rank test. Predictors of nonusage attrition (user group, sociodemographics, engagement parameters, and steps per day) were examined within univariate Cox proportional hazard regression. Predictors that had a univariate *P* value of <.25 [34] were selected for inclusion in a multivariate model. Hazard ratios, which represent relative risks for attrition, were calculated. Statistical analysis was performed with Stata version 12 (StataCorp LP, College Station, TX, USA).

## Results

### Overview

Of 16,040 users who answered the “Where did you hear about us?” question, most users (73.99%, 11,868/16,040) indicated that they heard about the program through their workplace. Further, 12.42% (1992/16,040) heard about 10,000 Steps from a friend; 4.3% (692/16,040) from a webpage; 2.9% (461/16,040) from a health professional; 0.9% (140/16,040) from Facebook; 0.41% (65/16,040) from other media including TV, newspaper, and radio; and 5.1% (822/16,040) indicated other sources.

### User Characteristics

Descriptions of the overall sample and the subsample regarding sociodemographics, engagement data, and logged physical activity are shown in Table 1 and Multimedia Appendix 1, respectively. The majority of participants in the overall sample were female (69.87%, 11,841/16,948) and Australian (77.54%, 13,142/16,948). Membership length ranged between 1 and 297 days (mean 190, SD 78.6 days). Gender and country of residence were significantly different between user groups, with highest percentage of females (73.5%, 942/1282) and Australians (84.7%, 1086/1282) for the Web-and-app group. App-only users and Web-and-app users were significantly younger than Web-only users (*P*<.001) with app-only users being also younger than Web-and-app users (*P*<.04). Web-only users (*P=*.004) and Web-and-app users (*P*<.001) had longer durations of membership in the 10,000 Steps program compared to the app-only users, with no difference between Web-only and Web-and-app users (*P*=.28).

**Table 1:** Sociodemographics, engagement, and physical activity by user group in the overall sample (N=16,948).

Variables		Overall N=16,948	Web only n=14,215	App only n=1451	Web and app n=1282	*F*_2,2_	χ^2^_2_	*P*
**Sociodemographics**								
	Age, mean (SD)	41.8 (12.1)	42.4 (12.2)	38.3 (11.1)	39.5 (11.5)	102.6		<.001^abc^
	Females, n (%)	11,841 (69.87)	9848 (69.28)	1051 (72.43)	942 (73.48)		14.8	<.001
	Australians, n (%)	13,142 (77.54)	10,936 (76.93)	1120 (77.19)	1086 (84.71)		41.0	<.001
	Membership days, mean (SD)	190.4 (78.6)	190.7 (78.9)	183.9 (79.6)	194.6 (72.7)	7.0		<.001^ac^
**Engagement**								
	Duration of usage (days), mean (SD)	34.5 (30.5)	32.8 (28.2)	37.7 (37.2)	50.2 (40.4)	203.6		<.001^abc^
	Individual challenges, mean (SD)	0.1 (0.5)	0.1 (0.4)	0.2 (0.7)	0.3 (0.9)	174.4		<.001^abc^
	Workplace challenges, mean (SD)	0.9 (0.5)	0.9 (0.5)	0.8 (0.7)	0.9 (0.6)	52.2		<.001^ac^
	Number of days physical activity was logged for, mean (SD)	30.8 (25.1)	29.5 (23.6)	32.9 (29.8)	43.3 (31.5)	188.7		<.001^abc^
**Physical activity**								
	Steps per day, mean (SD)	10,692.1 (4194.4)	10,701.7 (4251.6)	10,253.0 (3951.1)	11,082.6 (3758.1)	13.6		<.001^abc^

^a^ Web only is different from app only.

^b^ Web only is different from Web and app.

^c^ App only is different from Web and app.

### Engagement With the Intervention

In the overall sample, users utilized the program between 1 and 296 days (mean 34.5, SD 30.5 days) with 6.51% (1103/16,948) and 81.89% (13,879/16,948) participating at least in 1 individual challenge (range 0-8) and 1 workplace challenge (range 0-8), respectively. Users logged on average 30.8 days of physical activity (range 1-290 days) with 97.77% (16,400/16,948) of users logging physical activity more than once. With increasing length of membership, the average duration of usage, the number of individual and workplace challenges, as well as the number of days physical activity was logged per week decreased (Table 2). For example, users who were members between 1 and 2 months (30-60 days) used the program a mean 4.1 (SD 2.4) days per week, whereas users with a membership of at least 9 months (278-297 days) used the program a mean 1.1 (SD 0.9) days per week.

All engagement parameters differed significantly across user groups (*P*<.001). App-only users showed a longer duration of usage (*P*<.001), a higher number of individual challenges (*P*<.001), a lower number of workplace challenges (*P*<.001), and a higher number of days they logged physical activity (*P*<.001) compared to Web-only users (Table 1). Compared to app-only and Web-only users, Web-and-app users had a longer duration of usage (*P*<.001), higher number of individual (*P*=.02 and *P*<.001, respectively) challenges, as well as a higher number of days they logged physical activity (*P*<.001). Regarding workplace challenges, Web-and-app users had higher numbers compared to app-only users (*P*<.001), but were not significantly different from Web-only users (*P*=.07).

**Table 2:** Mean engagement parameters for different membership lengths in the overall sample (N=16,948).

Membership length	n	Duration of platform usage (days)		Individual challenges		Workplace challenges		Days physical activity logged	
		Mean (SD)	Mean/week^a^(SD)	Mean (SD)	Mean/week^a^(SD)	Mean (SD)	Mean/week^a^(SD)	Mean (SD)	Mean/week^a^(SD)
≤1 week (1-7 days)	54	3.6 (2.7)	10.1^b^(10.7)	0.00 (0.00)	0.00 (0.00)	0.24 (0.43)	0.59 (1.46)	3.5 (2.6)	9.9^b^ (10.6)
1-2 weeks (8-14 days)	60	9.0 (5.5)	5.5 (3.4)	0.10 (0.35)	0.07 (0.24)	0.60 (0.49)	0.37 (0.33)	8.7 (5.3)	5.4 (3.2)
2-3 weeks (15-21 days)	234	13.0 (6.1)	5.1 (2.4)	0.09 (0.09)	0.00 (0.04)	0.87 (0.46)	0.34 (0.18)	12.5 (5.9)	4.9 (2.3)
3-4 weeks (22-29 days)	413	15.7 (7.7)	4.6 (2.3)	0.04 (0.22)	0.01 (0.06)	0.85 (0.48)	0.25 (0.15)	14.0 (8.0)	4.1 (2.3)
1-2 months (30-60 days)	874	23.8 (14.4)	4.1 (2.4)	0.09 (0.35)	0.01 (0.06)	0.63 (0.49)	0.11 (0.09)	22.5 (14.1)	3.8 (2.4)
2-3 months (61-91 days)	1592	34.1 (18.7)	3.2 (1.8)	0.11 (0.41)	0.01 (0.04)	0.89 (0.50)	0.08 (0.04)	31.7 (17.8)	3.0 (1.7)
3-4 months (92-122 days)	604	36.2 (28.9)	2.4 (1.9)	0.26 (0.73)	0.02 (0.05)	0.66 (0.59)	0.04 (0.04)	32.8 (26.9)	2.2 (1.8)
4-5 months (123-153 days)	370	38.7 (32.5)	1.9 (1.6)	0.19 (0.67)	0.01 (0.03)	0.71 (0.65)	0.04 (0.03)	33.5 (28.6)	1.7 (1.4)
5-6 months (154-184 days)	1335	35.0 (34.0)	1.4 (1.4)	0.12 (0.56)	0.01 (0.02)	0.87 (0.53)	0.03 (0.02)	29.2 (24.5)	1.2 (1.0)
6-7 months (185-215 days)	3218	31.6 (27.9)	1.1 (1.0)	0.10 (0.47)	0.00 (0.02)	0.92 (0.37)	0.03 (0.01)	28.2 (22.7)	1.0 (0.8)
7-8 months (216-246 days)	4016	36.7 (34.1)	1.1 (1.0)	0.12 (0.61)	0.00 (0.02)	0.93 (0.65)	0.03 (0.02)	32.7 (27.5)	1.0 (0.8)
8-9 months (247-277 days)	2462	36.8 (30.5)	1.0 (0.8)	0.09 (0.49)	0.00 (0.01)	0.91 (0.46)	0.02 (0.01)	33.4 (26.0)	0.9 (0.7)
9-10 months (278-297 days)	1716	45.1 (37.4)	1.1 (0.9)	0.08 (0.42)	0.00 (0.01)	1.0 (0.58)	0.02 (0.01)	38.9 (30.4)	0.9 (0.7)

^a^ Calculated by (number of days respectively challenges / individual membership days)*7.

^b^ Numbers exceed 7 days (maximum membership length in this group) due to the opportunity to retrospectively log steps before registration.

### Recorded Physical Activity

More than half of participants (53.30%, 9033/16,948) logged at least 10,000 steps on average per day. However, app-only users had significantly lower numbers of steps per day compared to Web-only and Web-and-app users (*P*<.001) with lower numbers of steps per day for Web-only users compared to Web-and-app users (*P=*.01). Web-and-app users logged a mean of 67.4% (SD 29.94) of their total steps through the app.

### Prediction of Engagement Parameters by Group

Table 3 shows results of 4 linear regression analyses regarding the prediction of engagement parameters by user group when controlling for sociodemographics, length of membership, and logged physical activity. Results align with data from Table 1: comparisons of the number of workplace challenges within user groups did not reach the threshold for a meaningful effect when using Web-only as reference category (B=–0.07, η^2^=.005 and B=0.01, η^2^=.000). Web-and-app users showed a longer duration of usage (B=0.16, η^2^=.026), more individual challenges (B=0.12, η =.014), and more days of physical activity logged (B=0.15, η^2^=.024) compared to Web-only users; however, comparisons between app-only and Web-only users regarding duration of usage, number of individual challenges, and physical activity log days did not reach the threshold for a meaningful effect (η^2^=.004, η^2^=.009, η^2^=.003, respectively).

**Table 3:** Linear regression analyses showing associations between engagement parameters and app-only and Web-and-app groups in comparison to Web only in the overall sample (N=16,948).

Dependent variables	App only^a^			Web and app^a^		
	B (SE)	*P*	η^2^	B (SE)	*P*	η^2^
Duration of usage	0.06 (0.82)	<.001	.004	0.16 (0.86)	<.001	.026
Individual challenges	0.10 (0.01)	<.001	.009	0.12 (0.02)	<.001	.014
Workplace challenges	–0.07 (0.02)	<.001	.005	0.01 (0.02)	.14	.000
Physical activity log days	0.06 (0.67)	<.001	.003	0.15 (0.71)	<.001	.024

^a^ Web only was used as reference category; analyses controlled for age, gender, country, length of membership, and physical activity logged.

### Nonusage Attrition

**Figure 1:**
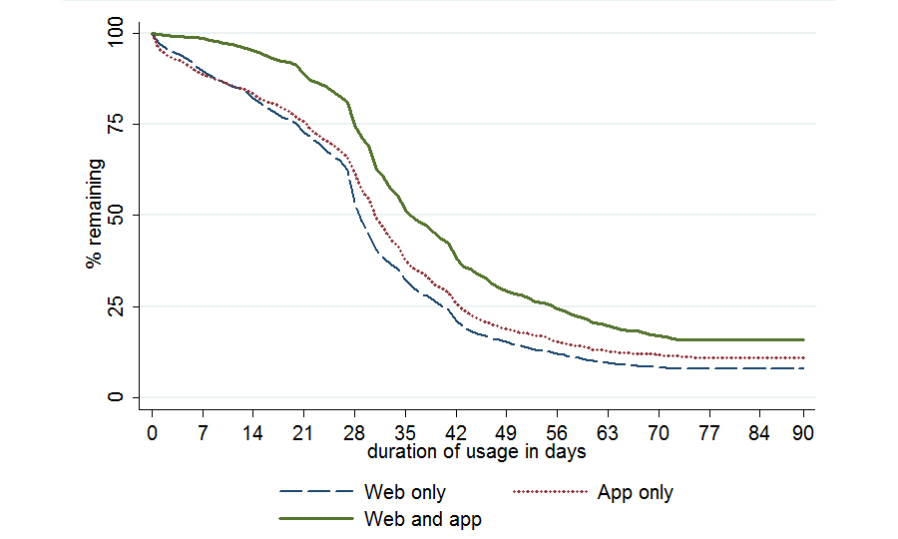
Nonusage attrition curves for user groups in the subsample of users who were 10,000 Steps members for at least 3 months (n=11,651).

The following results are based on a subsample only including users that had been a 10,000 Steps member for at least 3 months (n=11,651). Figure 1 presents Kaplan-Meier survival curves for the different user groups based on the duration of usage. The log-rank test showed that the survivor functions were significantly different across groups (χ^2^_2_=161.3, *P*<.001). Estimated median lifetime usage (time after which 50% stopped logging physical activity) was 30 days for all groups combined (Table 4). For all groups combined, 25.00% (2913/11,651) were still logging steps after 42 days. This was similar to the Web-only and app-only groups, with 41 and 43 days. respectively; however, in the Web-and-app group, 25.0% (220/878) of the sample were still logging steps after 56 days.

**Table 4:** Survival time by group in the subsample of users who were 10,000 Steps members for at least 3 months (n=11,651).

User group	Percentage of group still used platform^a^		
	75%	50%	25%
Web only	21 days	29 days	41 days
App only	22 days	31 days	43 days
Web and app	28 days	36 days	56 days
All users	21 days	30 days	42 days

^a^Table indicates at what point in time (days) 75%, 50%, and 25% of users were still using the platform for the different groups.

### Predictors of Nonusage Attrition

User groups, sociodemographics, engagement, and physical activity as potential predictors of nonusage attrition in the subsample are presented in Table 5. In the univariate analysis, app-only and Web-and-app users showed reduced attrition risk compared to Web-only users (hazard ratio=0.86, 95% CI 0.58-0.68, *P*<.001 and hazard ratio=0.63, 95% CI 0.81-0.93, *P*<.001, respectively). Being a resident of a country other than Australia (hazard ratio=0.87, 95% CI 0.82-0.91, *P*<.001), being male (hazard ratio=0.85, 95% CI 0.82-0.89, *P*<.001), and being of higher age (hazard ratio=0.992, 95% CI 0.991-0.994, *P*<.001) also reduced the risk of attrition. Regarding the engagement parameters, the risk of attrition decreased when the number of individual challenges (hazard ratio=0.62, 95% CI 0.59-0.66, *P*<.001), workplace challenges (hazard ratio=0.94, 95% CI 0.90-0.97, *P*<.001), and number of days physical activity was logged for (hazard ratio=0.921, 95% CI 0.919-0.922, *P*<.001) increased. Furthermore, the more steps logged per day lowered the risk for attrition (hazard ratio=0.99999, 95% CI 0.99998-0.99999, *P*<.001). All variables that had a *P*<.25 in the univariate analysis were included in the multivariate analysis. Results from the multivariate analysis showed a similar pattern (Table 5) except that app-only users did not differ from Web-only users (hazard ratio=0.98, 95% CI 0.91-1.05, *P*=.59) and there was no effect of workplace challenges on attrition risk (hazard ratio=0.98, 95% CI 0.94-1.01, *P=*.19) when controlling for other variables in the analysis.

**Table 5:** Univariate and multivariate Cox regression: association of nonusage attrition risk with user groups, sociodemographics, engagement, and physical activity in the subsample of users who were 10,000 Steps members for at least 3 months (n=11,651).

Dependent variables		Univariate		Multivariate	
		Hazard ratio (SE), 95% CI	*P*	Hazard ratio (SE), 95% CI	*P*
**Group**					
	Web only	reference		reference	
	App only	0.86 (0.03), 0.58-0.68	<.001	0.98 (0.04), 0.91-1.05	.59
	Web and app	0.63 (0.03), 0.81-0.93	<.001	0.69 (0.03), 0.64-0.75	<.001
**Country**					
	Australia	reference		reference	
	Other	0.87 (0.02), 0.82-0.91	<.001	0.55 (0.02), 0.52-0.58	<.001
**Gender**					
	Female	reference		reference	
	Male	0.85 (0.02), 0.82-0.89	<.001	0.95 (0.02), 0.91-0.99	.02
Age		0.992 (0.001), 0.991-0.994	<.001	0.999 (0.00), 0.997-1.000	<.001
Individual challenges		0.62 (0.02), 0.59-0.66	<.001	0.83 (0.03), 0.77-0.89	<.001
Workplace challenges		0.94 (0.02), 0.90-0.97	<.001	0.98 (0.02), 0.94-1.01	.19
Number of days physical activity was logged for		0.921 (0.001), 0.919-0.922	<.001	0.917 (0.001), 0.915-0.918	<.001
Steps per day		0.99999 (0.00000), 0.99998-0.99999	<.001	0.999980 (0.000002), 0.999976-0.999985	<.001

## Discussion

### Principal Findings

The aim of the present study was to examine program engagement with a freely accessible Internet-delivered physical activity intervention (10,000 Steps Australia) and test for a possible positive effect of using a smartphone app on engagement parameters and attrition. Results indicate a high program engagement and that the use of the app alone or in addition to the website can enhance program engagement and lower attrition. Further, this study extends previous research on individual challenges [20] by showing that workplace challenges were also associated with a prolonged usage of the program (reduced attrition risk).

### Program Engagement in Real World Compared to Controlled Settings

The present study reported high levels of program engagement. Nearly all (97%) users logged physical activity at least twice. Whereas most users engaged in at least 1 workplace challenge (81%), only 7% used individual challenges.

Although some studies conducted within controlled settings reported higher program engagement and lower attrition than the 10,000 Steps program [15,35], studies frequently report lower program engagement and higher attrition [36-40]. For example, Funk et al [35] reported a median of 124 exercise logs over 28 months (4.43 logs per months) within their weight loss maintenance program, whereas users from our study with a membership length of at least 9 months (278-297 days) had a median number of physical activity logs of 32 (3.33 logs per months). A study by Steele et al [39] reported 0.98 log-ins per week over 3 months, whereas our study recorded 3.0 physical activity logs per week for users with a membership length between 2 and 3 months. This is unexpected because all these studies were conducted in controlled settings, whereas our study was not. Studies from controlled trials are generally expected to have better outcomes regarding program usage because participants are likely to be more motivated and committed to the study because of the formal structure and selection process they went through compared to participants from noncontrolled settings [5,6]. Within controlled studies, it may be that the environment where the intervention was delivered plays an important role. Funk et al [35] provided their intervention within primary care clinics; this may have led to a higher commitment to the intervention. In contrast, the other studies recruited participants via local media advertising or email invitations; this provides a less structured environment that may make attrition easier [36-40].

### Program Engagement of 10,000 Steps Compared to Other Freely Accessible Programs

There has been some research examining engagement with Web-based interventions in real-life settings including interventions on depression, panic disorder, weight loss, physical activity, drinking behavior, and smoking [15-17,41-46]. Results of this study indicate a high program engagement compared to other freely accessible Web-based interventions. Whereas 97% of users in our study logged physical activity at least 2 times, other freely accessible studies reported between 10% and 62% visiting the intervention at least twice [15,42,43,45,46]. In a previous study on 10,000 Steps, a mean of physical activity logs per week of 1.6 was reported for a study period of 24 months [20]. This is higher than the mean in our study (0.9 logs/week over 9-10 months). This difference is likely caused by the selected sample in that study [20] because participants were users who already used the program for at least 1 month before recruitment and responded to an email invitation. Thus, participants were likely to be more motivated than in our study in which no such selection bias was present. Regarding nonusage attrition, our study showed longer usage of the program compared to other freely accessible interventions. Wanner et al [15] reported a median lifetime usage of 0 days for their physical activity website and after 1 month, only 7% of the registered users were still using the program. Also, Farvolden et al [44] reported only approximately 1% completed their 12-week open-access panic prevention program and Linke et al [41] reported only 24% of users remained in the intervention after 4 weeks for their sensible drinking program. Nevertheless, a commercial Web-based weight loss program [17] showed lower attrition for 12-week subscribers (median lifetime usage of 9 weeks) compared to our data. However, because users had to pay a subscription fee upfront, this have may led to a higher commitment to the intervention compared with studies that are free of any charge [5].

### Influence of Smartphone App on Engagement and Nonusage Attrition

The second aim of this study was to examine the effect of a smartphone app on program engagement. In general, app users were younger and more likely to be female compared to Web-only users, which aligns with research showing that the percentage of smartphone owners decreased with age and that young adults are more likely to use health apps [47]. Our results indicate that a smartphone app may assist in prolonging user engagement because using the 10,000 Steps app in addition to the website was associated with a longer duration of usage, higher participation in individual challenges, and higher number of days logging physical activity compared to users who only used the website. Further, nonusage attrition significantly differed across groups; the risk of nonusage attrition was reduced by using the app compared to only using the website to log steps. This effect was more pronounced for Web-and-app users with a 37% smaller attrition hazard compared to app-only users with a 14% smaller attrition hazard when using Web-only users as a reference category. These results are in-line with data from the case-matched control trial of the 10,000 Steps app showing positive effects of the app on the number of steps logged and days physical activity was logged for [27]. Previous research on the influence of smartphone apps on engagement and attrition are scarce. However, for their weight loss promotion app, Carter et al [25] found higher engagement and retention compared to the website diary. In accordance to our study, app users more often logged dietary records compared to the website group (92 days vs 35 days over 6 months). Overall, using smartphones for assistance in health promotion seems appealing because the percentage of people accessing health information via mobile devices is increasing. The Pew mobile health survey [47] found that approximately 52% of smartphone owners reported using their phone to look for health information and 19% have downloaded an app specifically to track or manage health.

Although our results are promising, it should be noted that the majority of users still were Web-only users (83.87%, 14,215/16,948) with only 16.13% (2733/16,948) using the app alone or in addition to the website. These uneven group sizes may be because the app is only available on the iOS mobile platform. Even though rates are increasing, in 2011 the proportion of US adults reporting to own either a smartphone or tablet was 50%, with 38% of smartphone owners and 52% of tablet owners saying their device used the iOS platform [48]. Thus, a substantial number of individuals had to use the website when interested in using the 10,000 Steps program because they either did not have a smartphone or had a mobile device running Android, Blackberry, or Windows. However, we cannot preclude that at least some users chose to use the website over the app intentionally (eg, because of a preference for browser-based surfing or reduced functionality of the app compared to the website).

### Sociodemographics, Engagement, and Physical Activity as Determinants of Nonusage Attrition

Personal factors associated with reduced nonusage attrition risk were being male, non-Australian, and older age. Differences in nonusage attrition by country of residency may be seen as an effect of weather because poor and extreme weather has been identified as barriers of physical activity [49] and, in Australia, it is hot and humid for most of the year. Even though evidence shows that females are more likely to be interested in health-related topics (eg, they are more likely to seek online for health information) [50], are more likely to participate in Web-based physical activity interventions [2,51], and use health apps on their phones more than men [47], in the univariate Cox regression males had a 15% smaller attrition hazard compared to females. However, some research suggests that men are more likely to participate in accelerometer-based studies [52]. Thus, men could be more attracted by technical devices as support for physical activity management (eg, pedometers used in the 10,000 Steps intervention) because they have a more positive attitude toward new technology [53] and, therefore, more interest in maintaining engagement with such an intervention. Effects of age on nonusage attrition are in-line with previous research showing that older age is associated with engagement with the intervention (eg, [45,46]) and as engagement increases, the risk of nonusage attrition decreases. As previous research on the 10,000 Steps project demonstrated [20], the number of individual challenges users participate in is associated with lower attrition risk. Further, this study adds evidence that workplace challenges reduce nonusage attrition risk. This aligns with previous research showing that interactive website components may promote engagement with the intervention [54].

### Implications for Future Research

Even though Web-based interventions are capable of reaching large parts of the population, a notable percentage never starts to use or accesses only a small part of the intervention [9]. Because content cannot be helpful if it is not viewed, techniques to enhance engagement with the intervention are needed. Previous research has identified factors that influence exposure and attrition in Internet-delivered interventions [19-22,54]. However, more research is needed to examine effects of such factors in real-life settings. For example, results from Wanner et al [15] suggested that reminder emails are only effective for trial participants, but not for registered open-access users. Previous research has identified peer support as a main facilitator of program engagement [21,54]. This is important because in our study the majority of users heard about the 10,000 Steps program either from their workplace or through a friend. Given the importance of social support as a mediator of behavior change, workplaces especially seem to be a valuable setting for physical activity promotion because its internal structure easily reaches large groups and provides a natural social network [55,56]. The majority of Web-based interventions we compared our results to also used interactive components including discussion boards or goal-setting features [35-38,44], such as the 10,000 Steps program does; however engagement was still higher in our study. This may be due to social support gained through doing workplace challenges within the 10,000 Steps program. This study provided evidence that within real-life settings the use of a smartphone app can enhance engagement with the intervention over time. Most studies report that overall engagement decreases over time (eg, [38]) and high attrition is widely seen as a challenge of Web-based interventions. However, some authors [17,57] argue that this is not necessarily a result of lost interest in the intervention, but of achieving a satisfactory level of behavior change or self-management skills [58]. Future research needs to target reasons for attrition and examine variables in experimental conditions that could distinguish people who decrease and increase engagement over time to design interventions that are likely to be used in long term.

### Limitations

A strength of this study is that we obtained a large sample from a freely available physical activity intervention and examined usability efficacy as recommended by previous literature [5]. However, there are some limitations that need to be considered when interpreting the results of this study. First, we only included users with at least 1 physical activity log within the period. Thus, engagement is likely to be higher compared with studies including users with 1 website log-in (people can log in to a website without using any features, such as the 10,000 Steps step log). On the other hand, usage duration was measured as days between first and last physical activity log. This may have underestimated engagement because users could have been active using the discussion board or competing in challenges while not logging steps. However, logging physical activity is the main feature of the 10,000 Steps program and represents a more credible measure than log-ins. Second, our sample included users with varying membership lengths and, therefore, different timeframes of actually being able to use the program. Because usage is likely to decline over time, study length has to be considered when comparing studies. Thus, we reported attrition only for users who were members for at least 3 months. However, we also reported results on engagement data for different membership lengths, which enables comparisons to previous research. Third, in this study we did not report on usage of the discussion forum and virtual walking buddies. This was because app-only users were not able to use these features in the same way as Web-only or Web-and-app users. The discussion forum is not accessible via the app at all; for the virtual walking buddies, users are not able to add buddies via the app. However, previous research has shown that the use of virtual walking buddies was positively associated with the average number of days physical activity was logged for [20]. Lastly, we did not measure physical activity in another form other than steps logged via the program. This is not an objective assessment of participants’ activity because logged steps do not necessarily encompass the overall physical activity level of the users. This study did not include an objective measure of physical activity assessing change from preregistration in the program. Although large accelerometer-based studies are emerging (eg, [59]), implementing such measures in the context of this study is challenging due to the timing of assessments. Withstanding their limitations, self-reported data for the period immediately before registering/commencing the program may provide a measure of physical activity that can be used to infer program efficacy in future studies.

### Conclusions

Our study provides insight into the engagement with a freely available physical activity intervention. Results indicate that smartphone apps may be powerful tools in enhancing program engagement and lower attrition. Future research should experimentally examine reasons of low engagement and attrition to enable development of interventions that ensure long-term engagement with the intervention. Further, our data elucidate that rating the success of online interventions by engagement parameters is highly dependent on the time window measured; therefore, study length has to be considered when comparing studies in regard to engagement parameters.

## Appendix

Multimedia Appendix 1 Sociodemographics, engagement, and physical activity by user group in the subsample of users who were 10,000 Steps members for at least 3 months (n=11,651).

## References

[1] (2014). World Health Organization.

[2] Davies CA, Spence JC, Vandelanotte C, Caperchione CM, Mummery WK (2012). Meta-analysis of internet-delivered interventions to increase physical activity levels. Int J Behav Nutr Phys Act.

[3] Joseph RP, Durant NH, Benitez TJ, Pekmezi DW (2014). Internet-based physical activity interventions. Am J Lifestyle Med.

[4] Bennett GG, Glasgow RE (2009). The delivery of public health interventions via the Internet: actualizing their potential. Annu Rev Public Health.

[5] Eysenbach G (2005). The law of attrition. J Med Internet Res.

[6] Kelders SM, Kok RN, Ossebaard HC, Van Gemert-Pijnen JE (2012). Persuasive system design does matter: a systematic review of adherence to web-based interventions. J Med Internet Res.

[7] Motl RW, Dlugonski D, Wójcicki TR, McAuley E, Mohr DC (2011). Internet intervention for increasing physical activity in persons with multiple sclerosis. Mult Scler.

[8] Glasgow RE, Christiansen SM, Kurz D, King DK, Woolley T, Faber AJ, Estabrooks PA, Strycker L, Toobert D, Dickman J (2011). Engagement in a diabetes self-management website: usage patterns and generalizability of program use. J Med Internet Res.

[9] Kelders SM, Van Gemert-Pijnen JE, Werkman A, Nijland N, Seydel ER (2011). Effectiveness of a Web-based intervention aimed at healthy dietary and physical activity behavior: a randomized controlled trial about users and usage. J Med Internet Res.

[10] Norman GJ, Zabinski MF, Adams MA, Rosenberg DE, Yaroch AL, Atienza AA (2007). A review of eHealth interventions for physical activity and dietary behavior change. Am J Prev Med.

[11] Donkin L, Christensen H, Naismith SL, Neal B, Hickie IB, Glozier N (2011). A systematic review of the impact of adherence on the effectiveness of e-therapies. J Med Internet Res.

[12] Enwald HP, Huotari ML (2010). Preventing the obesity epidemic by second generation tailored health communication: an interdisciplinary review. J Med Internet Res.

[13] Christensen H, Griffiths KM, Farrer L (2009). Adherence in Internet interventions for anxiety and depression. J Med Internet Res.

[14] Glasgow RE, Vogt TM, Boles SM (1999). Evaluating the public health impact of health promotion interventions: the RE-AIM framework. Am J Public Health.

[15] Wanner M, Martin-Diener E, Bauer G, Braun-Fahrländer C, Martin BW (2010). Comparison of trial participants and open access users of a web-based physical activity intervention regarding adherence, attrition, and repeated participation. J Med Internet Res.

[16] Christensen H, Griffiths KM, Korten AE, Brittliffe K, Groves C (2004). A comparison of changes in anxiety and depression symptoms of spontaneous users and trial participants of a cognitive behavior therapy website. J Med Internet Res.

[17] Neve MJ, Collins CE, Morgan PJ (2010). Dropout, nonusage attrition, and pretreatment predictors of nonusage attrition in a commercial Web-based weight loss program. J Med Internet Res.

[18] Hutchesson MJ, Collins CE, Morgan PJ, Callister R (2013). An 8-week web-based weight loss challenge with celebrity endorsement and enhanced social support: observational study. J Med Internet Res.

[19] Schneider F, de Vries H, Candel M, van de Kar A, van Osch L (2013). Periodic email prompts to re-use an internet-delivered computer-tailored lifestyle program: influence of prompt content and timing. J Med Internet Res.

[20] Davies C, Corry K, Van Itallie A, Vandelanotte C, Caperchione C, Mummery WK (2012). Prospective associations between intervention components and website engagement in a publicly available physical activity website: the case of 10,000 Steps Australia. J Med Internet Res.

[21] Richardson CR, Buis LR, Janney AW, Goodrich DE, Sen A, Hess ML, Mehari KS, Fortlage LA, Resnick PJ, Zikmund-Fisher BJ, Strecher VJ, Piette JD (2010). An online community improves adherence in an internet-mediated walking program. Part 1: results of a randomized controlled trial. J Med Internet Res.

[22] Lewis B, Williams D, Dunsiger S, Sciamanna C, Whiteley J, Napolitano M, Bock B, Jakicic J, Getz M, Marcus B (2008). User attitudes towards physical activity websites in a randomized controlled trial. Prev Med.

[23] Brouwer W, Oenema A, Raat H, Crutzen R, de Nooijer J, de Vries NK, Brug J (2010). Characteristics of visitors and revisitors to an Internet-delivered computer-tailored lifestyle intervention implemented for use by the general public. Health Educ Res.

[24] Bort-Roig J, Gilson ND, Puig-Ribera A, Contreras RS, Trost SG (2014). Measuring and influencing physical activity with smartphone technology: a systematic review. Sports Med.

[25] Carter MC, Burley VJ, Nykjaer C, Cade JE (2013). Adherence to a smartphone application for weight loss compared to website and paper diary: pilot randomized controlled trial. J Med Internet Res.

[26] Cobb NK, Poirier J (2014). Effectiveness of a multimodal online well-being intervention: a randomized controlled trial. Am J Prev Med.

[27] Kirwan M, Duncan MJ, Vandelanotte C, Mummery WK (2012). Using smartphone technology to monitor physical activity in the 10,000 Steps program: a matched case-control trial. J Med Internet Res.

[28] Brown WJ, Mummery K, Eakin EG, Schofield G (2006). 10,000 Steps Rockhampton: Evaluation of a whole community approach to improving population levels of physical activity. J Phys Act Health.

[29] Antypas K, Wangberg SC (2014). An Internet- and mobile-based tailored intervention to enhance maintenance of physical activity after cardiac rehabilitation: short-term results of a randomized controlled trial. J Med Internet Res.

[30] Tudor-Locke C, Ham SA, Macera CA, Ainsworth BE, Kirtland KA, Reis JP, Kimsey CD (2004). Descriptive epidemiology of pedometer-determined physical activity. Med Sci Sports Exerc.

[31] De Cocker KA, De Bourdeaudhuij IM, Brown WJ, Cardon GM (2007). Effects of "10,000 steps Ghent": a whole-community intervention. Am J Prev Med.

[32] Cohen J (1988). Statistical Power Analysis for the Behavioral Sciences.

[33] Ware LJ, Hurling R, Bataveljic O, Fairley BW, Hurst TL, Murray P, Rennie KL, Tomkins CE, Finn A, Cobain MR, Pearson DA, Foreyt JP (2008). Rates and determinants of uptake and use of an internet physical activity and weight management program in office and manufacturing work sites in England: cohort study. J Med Internet Res.

[34] Hosmer DW, Lemeshow S, Sturdivant RX (2013). Model-building strategies and methods for logistic regression. Applied Logistic Regression.

[35] Funk KL, Stevens VJ, Appel LJ, Bauck A, Brantley PJ, Champagne CM, Coughlin J, Dalcin AT, Harvey-Berino J, Hollis JF, Jerome GJ, Kennedy BM, Lien LF, Myers VH, Samuel-Hodge C, Svetkey LP, Vollmer WM (2010). Associations of Internet website use with weight change in a long-term weight loss maintenance program. J Med Internet Res.

[36] Danaher BG, Boles SM, Akers L, Gordon JS, Severson HH (2006). Defining participant exposure measures in Web-based health behavior change programs. J Med Internet Res.

[37] Brindal E, Freyne J, Saunders I, Berkovsky S, Smith G, Noakes M (2012). Features predicting weight loss in overweight or obese participants in a web-based intervention: randomized trial. J Med Internet Res.

[38] McKay HG, King D, Eakin EG, Seeley JR, Glasgow RE (2001). The diabetes network internet-based physical activity intervention: a randomized pilot study. Diabetes Care.

[39] Steele R, Mummery WK, Dwyer T (2007). Using the Internet to promote physical activity: a randomized trial of intervention delivery modes. J Phys Act Health.

[40] Hurling R, Catt M, Boni MD, Fairley BW, Hurst T, Murray P, Richardson A, Sodhi JS (2007). Using Internet and mobile phone technology to deliver an automated physical activity program: randomized controlled trial. J Med Internet Res.

[41] Linke S, Murray E, Butler C, Wallace P (2007). Internet-based interactive health intervention for the promotion of sensible drinking: patterns of use and potential impact on members of the general public. J Med Internet Res.

[42] Wang J, Etter J (2004). Administering an effective health intervention for smoking cessation online: the international users of Stop-Tabac. Prev Med.

[43] Balmford J, Borland R, Benda P (2008). Patterns of use of an automated interactive personalized coaching program for smoking cessation. J Med Internet Res.

[44] Farvolden P, Denisoff E, Selby P, Bagby RM, Rudy L (2005). Usage and longitudinal effectiveness of a Web-based self-help cognitive behavioral therapy program for panic disorder. J Med Internet Res.

[45] Lara MA, Tiburcio M, Aguilar AA, Sánchez-Solís A (2014). A four-year experience with a Web-based self-help intervention for depressive symptoms in Mexico. Rev Panam Salud Publica.

[46] Verheijden MW, Jans MP, Hildebrandt VH, Hopman-Rock M (2007). Rates and determinants of repeated participation in a web-based behavior change program for healthy body weight and healthy lifestyle. J Med Internet Res.

[47] Fox S, Duggan M (2012). Mobile Health 2012.

[48] (2012). The Future of Mobile News: The Explosion in Mobile Audiences and a Close Look at What it Means for News.

[49] Tucker P, Gilliland J (2007). The effect of season and weather on physical activity: a systematic review. Public Health.

[50] Fox S, Duggan M (2013). Health Online 2013.

[51] Vandelanotte C, Spathonis KM, Eakin EG, Owen N (2007). Website-delivered physical activity interventions a review of the literature. Am J Prev Med.

[52] Harris TJ, Victor CR, Carey IM, Adams R, Cook DG (2008). Less healthy, but more active: opposing selection biases when recruiting older people to a physical activity study through primary care. BMC Public Health.

[53] Schumacher P, Morahan-Martin J (2001). Gender, Internet and computer attitudes and experiences. Computers in Human Behavior.

[54] Brouwer W, Kroeze W, Crutzen R, de Nooijer J, de Vries NK, Brug J, Oenema A (2011). Which intervention characteristics are related to more exposure to internet-delivered healthy lifestyle promotion interventions? A systematic review. J Med Internet Res.

[55] Lauzon N, Chan CB, Myers AM, Tudor-Locke C (2008). Participant experiences in a workplace pedometer-based physical activity program. J Phys Act Health.

[56] Robroek SJ, van Lenthe FJ, van Empelen P, Burdorf A (2009). Determinants of participation in worksite health promotion programmes: a systematic review. Int J Behav Nutr Phys Act.

[57] Ritterband LM, Thorndike FP, Cox DJ, Kovatchev BP, Gonder-Frederick LA (2009). A behavior change model for internet interventions. Ann Behav Med.

[58] Bandura A (2005). The primacy of self-regulation in health promotion. Applied Psychology.

[59] Troiano RP, Berrigan D, Dodd KW, Mâsse LC, Tilert T, McDowell M (2008). Physical activity in the United States measured by accelerometer. Med Sci Sports Exerc.

