# Supplementary material for: Engagement and Nonusage Attrition With a Free Physical Activity Promotion Program: The Case of 10,000 Steps Australia
Source: J Med Internet Res. 2015 Jul 15;17(7):e176. doi: 10.2196/jmir.4339 (PMC4526999; doi:10.2196/jmir.4339)
Supplement: Multimedia Appendix 1 [file jmir_v17i7e176_app1.pdf]

Supplementary Table 1. Sociodemographics, engagement, and physical activity by user group in the subsample of users who were 10,000 Steps members for at least 3 months (n=11,651).

Variables	Overall n=11,651	Web only n=9800	App only n=973	Web and app n=878	$F_{2,2}$	χ^2_2	*P*
**Sociodemographics**							
Age, mean (SD)	41.4 (11.9)	41.9 (12.0)	38.3 (11.2)	38.7 (11.1)	66.0		<.001 [ab]
Females, n (%)	8266 (70.95)	6920 (70.61)	703 (72.3)	643 (73.2)		3.7	.17
Australians, n (%)	9631 (82.66)	8001 (81.64)	841 (86.4)	789 (89.9)		48.5	<.001
Membership days, mean (SD)	223.2 (44.7)	223.6 (44.7)	219.5 (45.8)	223.0 (43.2)	3.8		.02 [a]
**Engagement**							
Duration of usage (days), mean (SD)	36.2 (30.3)	34.4 (28.0)	40.2 (36.7)	51.0 (40.8)	132.5		<.001 [abc]
Individual challenges, mean (SD)	0.1 (0.5)	0.1 (0.4)	0.2 (0.7)	0.3 (0.9)	119.7		<.001 [abc]
Workplace challenges, mean (SD)	0.9 (0.5)	0.9 (0.5)	0.9 (0.6)	1.0 (0.5)	9.25		<.001 [abc]
Number of days physical activity was logged for, mean (SD)	32.3 (24.6)	31.0 (23.3)	34.9 (28.4)	43.4 (30.7)	110.3		<.001 [abc]
**Physical activity**							
Steps per day, mean (SD)	10,869.1 (4122.7)	10,853.1 (4184.3)	10,586.3 (3789.0)	11,361.5 (3728.6)	8.6		<.001 [bc]

[a] Web only is different from app only.

[b] Web only is different from Web and app.

[c] App only is different from Web and app.
